# Supplementary material for: A Likelihood Approach for Real-Time Calibration of Stochastic Compartmental Epidemic Models
Source: PLoS Comput Biol. 2017 Jan 17;13(1):e1005257. doi: 10.1371/journal.pcbi.1005257 (PMC5240920; doi:10.1371/journal.pcbi.1005257)
Supplement: S1 File — (TAR.GZ) [file pcbi.1005257.s014.tar.gz › HSPH_Online-SI-Revision/Additions/PF09/n100-extreme-SEITR/n100-extreme-SEITR_PF_summary.pdf]

peak −8	R0	$1/\theta_2$	inf	inflog	newly	newlylog	Reff	AttackRate
mean	26.3	52.3	993.4	0.6	485.4	0.5	26.2	24.
median	25.3	43.4	199.	0.4	76.9	0.3	25.6	22.4
Quantile .1	12.5	11.3	34.8	0.2	34.4	0.2	11.8	16.3
Quantile .9	35.6	102.9	3141.1	1.3	1485.9	1.1	35.	35.5
36	29	2						
length	50							

peak −4	R0	$1/\theta_2$	inf	inflog	newly	newlylog	Reff	AttackRate
mean	38.7	59.5	33.8	0.1	44.1	0.3	43.5	5.6
median	26.4	47.4	26.7	0.1	43.5	0.2	31.4	3.3
Quantile .1	6.2	9.2	5.2	0.	24.8	0.1	14.3	0.7
Quantile .9	82.7	125.4	62.2	0.3	67.6	0.5	87.	14.5
7	2	6						
length	50							

peak	R0	$1/\theta_2$	inf	inflog	newly	newlylog	Reff	AttackRate
mean	40.8	71.1	29.2	0.1	37.3	0.2	470.8	4.6
median	29.4	60.1	22.8	0.1	35.5	0.2	371.	3.5
Quantile .1	6.6	9.4	7.8	0.	21.8	0.1	217.4	0.5
Quantile .9	82.7	178.6	57.8	0.3	55.7	0.4	776.8	9.3
0	0	1						
length	50							

peak +4	R0	$1/\theta_2$	inf	inflog	newly	newlylog	Reff	AttackRate
mean	40.8	71.1	345.3	0.5	167.5	0.4	1091.8	4.6
median	29.4	60.1	196.7	0.5	97.7	0.3	937.6	3.5
Quantile .1	6.6	9.4	32.4	0.1	33.8	0.1	268.1	0.5
Quantile .9	82.7	178.6	963.8	1.	472.9	0.8	2323.	9.6
0	0	0						
length	50							

peak +8	R0	$1/\theta_2$	inf	inflog	newly	newlylog	Reff	AttackRate
mean	40.8	71.1	2621.1	1.	1222.3	0.9	865.9	4.6
median	29.4	60.	739.4	0.9	507.8	0.8	787.9	3.4
Quantile .1	6.7	9.4	116.8	0.3	113.1	0.3	164.	0.5
Quantile .9	82.7	178.6	6499.7	1.8	3591.	1.6	1445.3	9.5
0	0	1						
length	50							
